# Case Report: Pleuro-myopericarditis in a frail older patient in an acute geriatric unit: the evidence gap in guideline-based therapy

**DOI:** 10.3389/fcvm.2026.1798612

**Published:** 2026-05-18

**Authors:** Giovanni Artuso, Antonella Risoli, Giorgio Ozino Caligaris, Daniele Pellerani, Letizia Alessandrini, Vittoria Ingrosso, Caterina Corcione, Francesca Casella, Elisabetta Serafini, Michele Ciaburri, Manuela Antocicco, Laura Gerardino, Ludovico Luca Sicignano, Elena Verrecchia, Federica D'Ignazio, Carla Recupero, Maria Modestina Bulla, Fiammetta Albi, Federica Re, Domenico Gabrielli, Francesco Landi, Giuseppe Zuccalà

**Affiliations:** 1 Dipartimento di Scienze dell'Invecchiamento, Ortopediche e Reumatologiche, IRCCS, Roma, Italia; 2 Dipartimento Cardio-Toraco-Vascolare, Azienda Ospedaliera San Camillo Forlanini, Rome, Italy

**Keywords:** acute pericarditis, comorbidities, frailty, myopericarditis, older adults, pericardial effusion (PE), pericarditis

## Abstract

**Introduction:**

Current ESC guidelines recommend NSAIDs and colchicine as first-line therapy for acute pericarditis. However, supporting evidence is largely derived from younger and clinically stable populations, with frail older patients underrepresented, highlighting a gap between guideline recommendations and real-world applicability.

**Case presentation:**

We report the case of an 87-year-old frail man with multiple comorbidities who developed pleuro-myopericarditis with moderate-to-severe pericardial effusion. First-line therapy with ibuprofen and colchicine was initiated but discontinued early due to severe gastrointestinal intolerance and worsening renal function. Pericardiocentesis was considered; however, according to current guideline recommendations, it is primarily indicated in the presence of cardiac tamponade or for selected diagnostic purposes. In this case, the absence of hemodynamic compromise and the high procedural risk related to frailty led to conservative management. A corticosteroid-based regimen was therefore initiated, resulting in clinical improvement and gradual reduction of pericardial effusion.

**Discussion:**

This case highlights the limitations of guideline-directed therapy in frail elderly patients, in whom comorbidities, renal dysfunction, and polypharmacy frequently limit the tolerability of NSAIDs and colchicine. It underscores the gap between guideline recommendations and real-world clinical applicability, emphasizing the need for individualized therapeutic strategies.

**Conclusion:**

Management of acute pericarditis in frail older adults should be individualized, integrating guideline recommendations with geriatric assessment and clinical judgment. Optimal treatment may differ from guideline-based strategies when patient-specific risks outweigh expected benefits. This case underscores the urgent need for evidence specifically addressing this growing and vulnerable population.

## 1 Introduction

According to the most recent guidelines issued by the European Society of Cardiology, first-line treatment for acute pericarditis consists of high-dose non-steroidal anti-inflammatory drugs (NSAIDs) in combination with colchicine, with a Class I recommendation and Level of Evidence A (1). This therapeutic approach has been shown to reduce symptom duration and significantly lower recurrence rates in the general population (2, 3). However, the evidence supporting these recommendations is largely derived from randomized clinical trials enrolling predominantly younger and clinically stable individuals (2, 3), whereas frail older adults are markedly underrepresented. Consequently, the generalizability of guideline-directed therapy to the geriatric population remains uncertain (4). In older patients, acute pericarditis frequently presents with atypical or nonspecific manifestations. While chest pain represents the predominant symptom in younger individuals, older adults more commonly present with dyspnea, functional decline, or general deterioration, which may delay diagnosis and complicate management (5, 6). In addition, age-related physiological changes affecting pharmacokinetics and pharmacodynamics (7), together with multimorbidity, chronic kidney disease, and polypharmacy, substantially increase the risk of adverse drug reactions and limit the tolerability of standard anti-inflammatory regimens (7, 8). Although guideline-recommended therapy remains the cornerstone of acute pericarditis management, its application in frail older patients often requires dose reduction, treatment modification, or discontinuation due to safety concerns. In this setting, individualized clinical judgment becomes essential. We report a case that highlights the challenges of applying ESC guideline recommendations in a frail older patient with acute pericarditis and discuss the implications for real-world clinical practice.

## 2 Case presentation

An 87-year-old man was admitted to the Geriatrics Unit for fever with shaking chills and an episode of oppressive chest discomfort. The patient presented with moderate to severe frailty (Clinical Frailty Scale = 6/9) and was partially independent in activities of daily living (ADL 4/6) and dependent in instrumental activities of daily living (IADL 2/8). He had recently been hospitalized in a rehabilitation facility following a right femoral fracture treated with hip prosthesis implantation. His medical history included rheumatoid arthritis, chronic anemia, arterial hypertension, hypothyroidism, chronic kidney disease, thoraco-abdominal aortic aneurysm surgically treated in 2022 and 2023, and a left popliteal aneurysm under follow-up. Chronic medications included antiplatelet therapy, statins, antihypertensives, levothyroxine, hydroxychloroquine, low-dose corticosteroids, and anticoagulant prophylaxis. Hydroxychloroquine therapy was continued throughout hospitalization. Baseline low-dose corticosteroid therapy was initially maintained and subsequently up-titrated when systemic corticosteroid treatment was introduced for pericarditis management. Diagnostic evaluation revealed electrocardiographic abnormalities consistent with myopericardial involvement.

**Figure 1:**
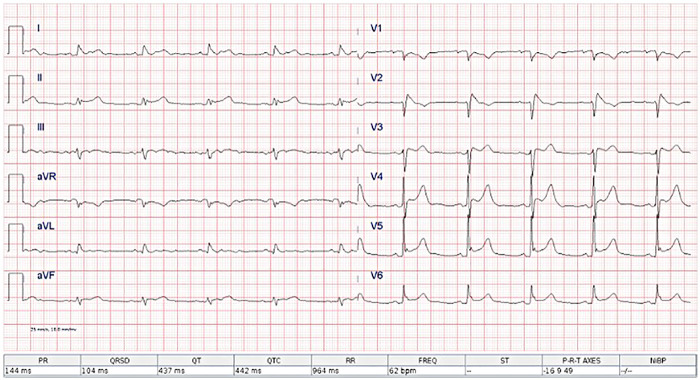
Twelve-lead electrocardiogram tracing shows regular rhythm at 62 beats per minute, with notable tall R waves in leads V4 through V6, and minor ST segment depression. PR, QRS, and QT intervals are listed below the tracing.

Diagnostic evaluation revealed electrocardiographic abnormalities consistent with myopericardial involvement. The 12-lead electrocardiogram demonstrated sinus rhythm at 62 beats per minute with normal atrioventricular conduction (PR interval 144 ms) and a narrow QRS complex (104 ms). The electrical axis was within normal limits. The corrected QT interval (QTc 442 ms) was at the upper limit of normal. Diffuse ST-segment elevation with a concave morphology was observed, predominantly in the anterior and lateral precordial leads (V2-V5), without reciprocal ST-segment depression. No pathological Q waves were present, and R-wave progression was preserved. T waves were positive and symmetric in the affected leads. Overall, the ECG pattern was more consistent with inflammatory myocardial-pericardial involvement rather than acute ST-segment elevation myocardial infarction (STEMI), given the absence of reciprocal changes and the morphology of ST-segment elevation. (Figure 1); Elevated inflammatory markers such as CRP 253.2 mg/L were found (Supplementary Table 1).

**Figure 2:**
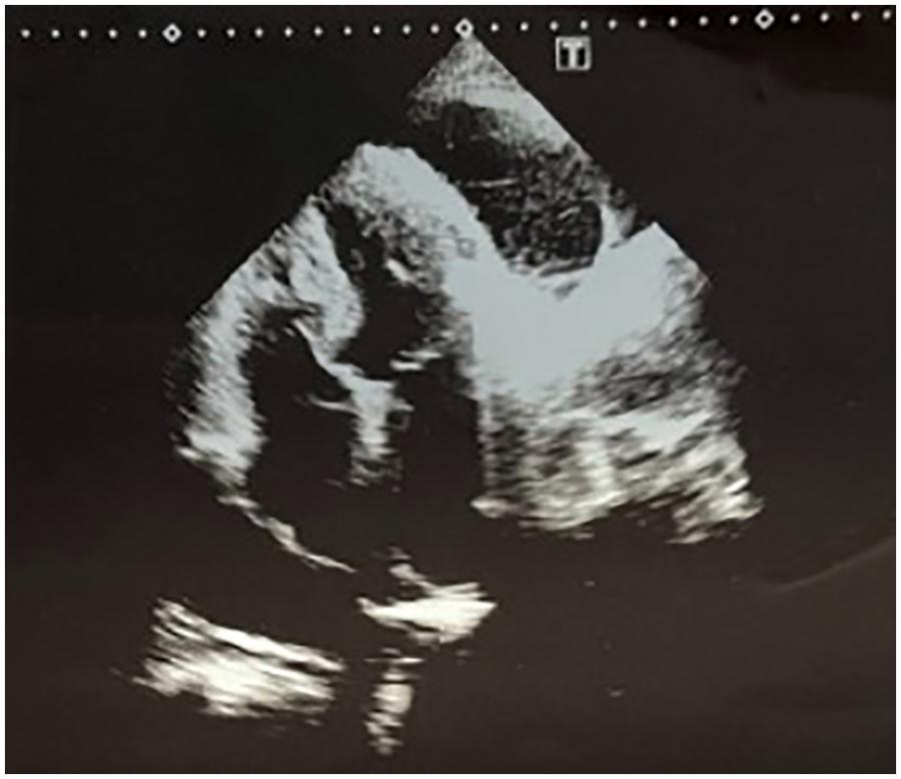
Echocardiogram image displays a fourchamber view of the heart showing detailed anatomical structures including ventricles and atria, with grayscale contrast representing tissue density and blood flow.

**Figure 3:**
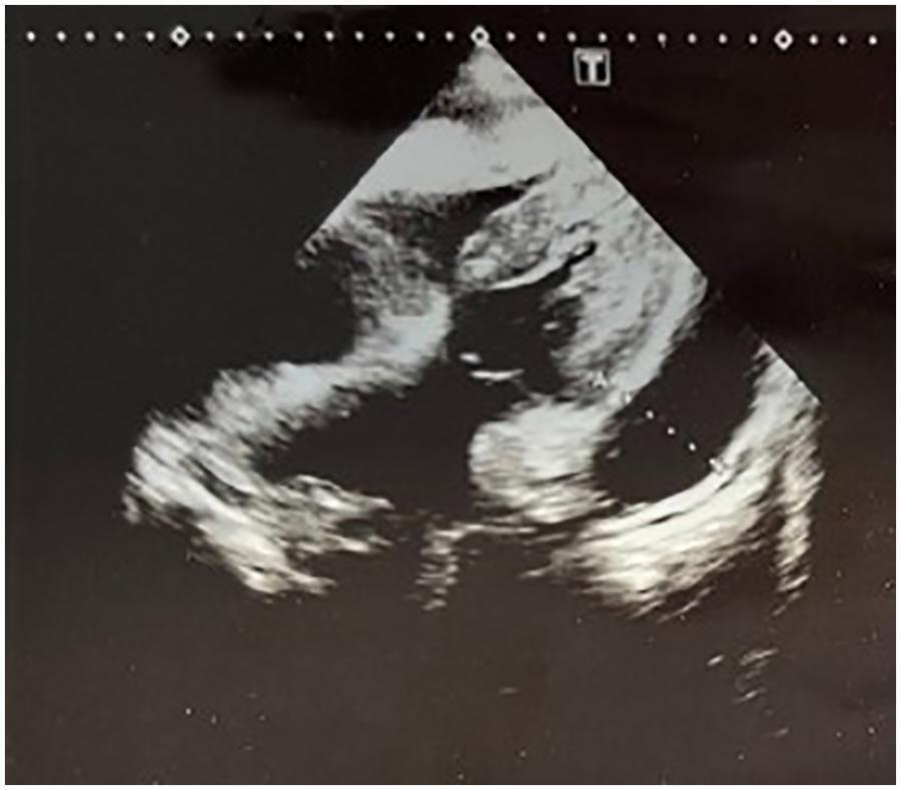
The subcostal window with appreciable moderate pericardial effusion at the level of the free wall of the left ventricle.

**Figure 4:**
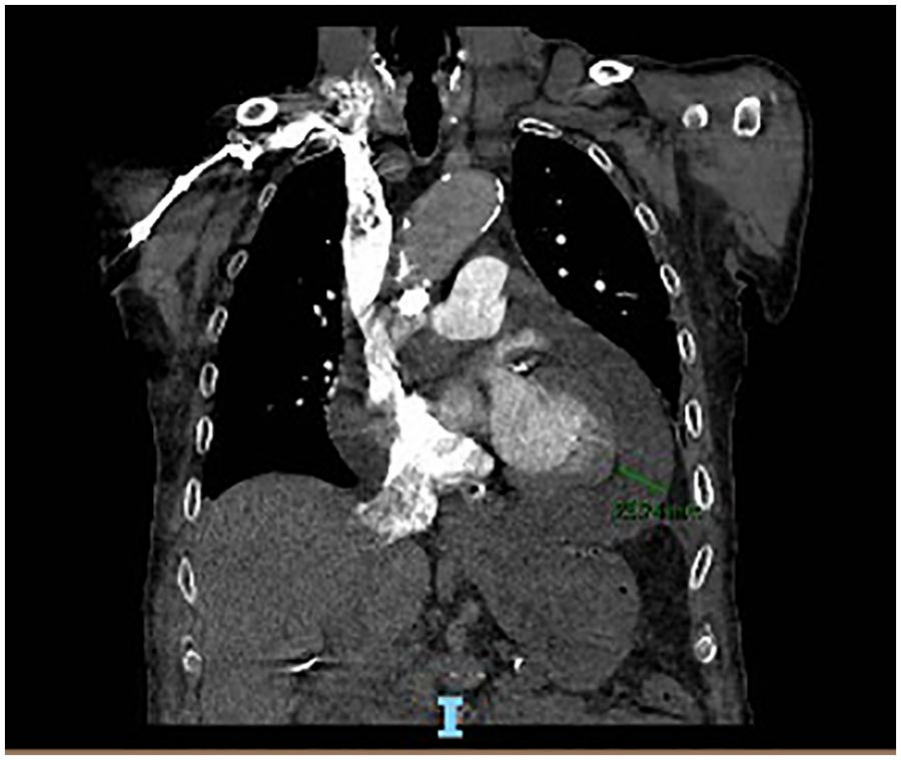
Coronal CT scan of the chest displaying the thoracic cavity, including lungs, heart, and adjacent structures, with significant white areas indicating abnormal tissue or mass along the mediastinum and left lung region.

Transthoracic echocardiography demonstrated a circumferential pericardial effusion, predominantly distributed along the posterior and lateral walls. The effusion appeared as an anechoic space separating the visceral and parietal pericardial layers, more evident in the parasternal and apical views. The maximum separation between pericardial layers was visually consistent with a moderate effusion, without echocardiographic signs of fibrinous organization or septations. No evidence of right atrial or right ventricular diastolic collapse was observed. Inferior vena cava size and collapsibility (as previously reported) did not suggest overt hemodynamic compromise at the time of examination. Left ventricular systolic function appeared preserved (Figures 2, 3). Contrast-enhanced chest CT demonstrated a moderate circumferential pericardial effusion (max thickness ∼20–23 mm), predominantly along the posterior and lateral left ventricular walls, without signs of pericardial thickening, loculation, or cardiac chamber compression. A concomitant moderate left-sided pleural effusion (∼22–23 mm) was observed, layering dependently in the basal pleural space, free-flowing and without septations. Mild adjacent compressive atelectasis was present (Figure 4). Furthermore, the predominantly posterolateral distribution of the pericardial effusion made pericardiocentesis technically less accessible and more challenging, thereby increasing procedural risk and supporting the decision for conservative management.

Overall, imaging findings were consistent with inflammatory pleuro-myopericardial involvement without radiological evidence of hemodynamic compromise. The patient also had myocardial involvement with an increase in troponin I Hs values from 461 to 35 ng/L. Cardiac MRI was not performed due to worsening clinical conditions and complexity. Autoimmunity was negative. No pericardial manipulation was performed, and in light of the clinical history, this case does not represent a case of delayed post-pericardiotomy syndrome.

Immobilisation can contribute to immune dysfunction, but it is not generally a primary cause of acute serous inflammation.

The event occurred approximately 20 days after hip replacement surgery; this time interval makes a simple postoperative systemic inflammatory response syndrome, which typically occurs within the first 48–72 h after surgical trauma, unlikely. Furthermore, the absence of cardiac surgery allows us to rule out a post-pericardiotomy syndrome.

Overall, the clinical picture appears to be more compatible with an inflammatory process secondary to systemic infectious aetiology, in light of the positive blood cultures, which are likely responsible for the pleuro-myopericardial involvement. In this context, the pathogenetic mechanism could be attributable to both direct infectious damage and a secondary immune response triggered by the pathogen. In this case the aetiology of pleuropericarditis was attributed to a post-surgical infectious cause, given the positive blood cultures for Staphylococcus aureus complex.

In accordance with ESC guideline recommendations, anti-inflammatory therapy was initiated with ibuprofen 600 mg three times daily and colchicine 0.5 mg twice daily. After approximately four days of treatment, the patient developed significant epigastric pain and worsening renal function. Despite dose reduction, gastrointestinal intolerance persisted, leading to complete discontinuation of NSAIDs and colchicine after less than one week of exposure.

Given the inability to continue first-line therapy, systemic corticosteroid treatment was initiated with prednisone at approximately 0.3 mg/kg/day and continued for several days. (Supplementary Table 2) The rationale for initiating systemic corticosteroid therapy was both an intolerance to colchicine despite a reduction in dosage and the use of full-dose gastroprotection since admission to the ward. This approach resulted in symptomatic improvement, slow reduction of the pericardial effusion and stabilization of renal function. We do not have any data regarding hospital follow-up, as the patient died during hospitalisation due to septic shock caused by a new nosocomial infection. Patient perspective could not be obtained due to clinical deterioration and subsequent death.

## 3 Discussion

This case highlights the challenges of managing acute pleuro-myopericarditis in frail older patients, in whom the applicability of guideline-directed therapy is often limited by comorbidities, reduced physiological reserve, and increased susceptibility to adverse drug reactions.

### 3.1 Limitations of guideline-based therapy in frail elderly patients

Current ESC guidelines recommend the combination of non-steroidal anti-inflammatory drugs (NSAIDs) and colchicine as first-line therapy for acute pericarditis, supported by high-level evidence (1). However, the clinical trials underpinning these recommendations have predominantly enrolled younger and clinically stable individuals (2, 3), with frail older adults being largely underrepresented. As a result, the external validity of guideline-based therapy in geriatric populations remains uncertain (4).

Frail older patients frequently present with multimorbidity, chronic kidney disease, and polypharmacy, all of which significantly influence both pharmacokinetics and pharmacodynamics (7, 8). In addition, age-related physiological changes and reduced homeostatic reserve increase vulnerability to drug toxicity (7). Consequently, strict adherence to guideline-recommended regimens may not be feasible in this population, and early discontinuation of treatment is common in real-world clinical practice.

This case exemplifies how guideline-directed therapy may be difficult to implement in frail patients, requiring prompt reassessment and adaptation of the therapeutic strategy based on individual tolerance and overall clinical context. This case adds to the limited literature on pericarditis management in frail elderly patients, highlighting the gap between guideline recommendations and real-world clinical applicability.

### 3.2 Risks associated with NSAIDs and colchicine

In frail older adults, the risk–benefit profile of NSAIDs and colchicine differs substantially from that observed in younger populations. NSAIDs are associated with well-established gastrointestinal and renal risks, which are amplified in the presence of chronic kidney disease, advanced age, and concomitant medications. Inhibition of cyclooxygenase leads to reduced prostaglandin synthesis, impairing gastric mucosal protection and predisposing to ulceration and bleeding (9). At the renal level, NSAID-induced reduction in prostaglandin-mediated vasodilation may precipitate acute kidney injury, particularly in patients receiving renin–angiotensin system inhibitors and diuretics, a combination known to markedly increase nephrotoxicity risk (10, 11).

Colchicine, although effective in reducing recurrence rates (2), requires careful dose adjustment in patients with impaired renal function. Reduced clearance may result in drug accumulation, increasing the risk of gastrointestinal intolerance, myopathy, and neuromuscular toxicity, especially in elderly and polymedicated individuals (12).

In the present case, the early onset of severe epigastric pain and worsening renal function led to discontinuation of both NSAIDs and colchicine despite dose reduction. This clinical scenario reflects a common real-world limitation of first-line therapy in frail older patients, where intolerance may preclude adequate treatment duration and effectiveness.

### 3.3 Alternative therapeutic strategies

When first-line therapy is not tolerated, alternative treatment strategies must be considered. Corticosteroids are generally regarded as second-line therapy due to their association with increased recurrence rates (1, 4); however, in selected frail patients, they may represent a pragmatic and clinically appropriate option. In patients already receiving chronic corticosteroid therapy, management of acute pericarditis may require dose escalation rather than initiation of *de novo* treatment. In this context, increasing the corticosteroid dose to an anti-inflammatory range (approximately 0.2–0.5 mg/kg/day of prednisone equivalent) is often necessary to achieve disease control. In frail elderly patients, lower starting doses may be considered based on tolerability and comorbidity burden. Once clinical improvement and reduction of inflammatory markers are achieved, gradual tapering should be implemented to minimize the risk of recurrence and steroid-related adverse effects. In our case, initiation of low-to-moderate-dose corticosteroid therapy resulted in clinical improvement, stabilization of renal function, and a gradual reduction in pericardial effusion.

More recently, interleukin-1 (IL-1) inhibitors such as anakinra and rilonacept have emerged as effective therapeutic options in recurrent and refractory pericarditis, targeting key inflammatory pathways. These agents have demonstrated rapid symptom control and steroid-sparing effects in clinical studies and real-world registries (13–15). The most recent ESC updates further support a growing role for IL-1 inhibition in selected patients (16). Nevertheless, evidence regarding their safety and efficacy in very old and frail patients remains limited, and their use in this population requires further investigation.

In addition, invasive strategies such as pericardiocentesis should be carefully evaluated on an individual basis. According to current guidelines, pericardiocentesis is primarily indicated for cardiac tamponade or for diagnostic purposes in selected cases (1). In the present case, despite a moderate-to-severe pericardial effusion and a suspected infectious aetiology, pericardiocentesis was deferred due to the absence of hemodynamic compromise and the high procedural risk associated with frailty and comorbidities. This decision underscores the importance of balancing potential diagnostic and therapeutic benefits against procedural risks in vulnerable patients.

This case report was prepared in accordance with the CARE (CAse REport) guidelines to ensure completeness and transparency of clinical reporting (17).

## 4 Conclusion

In frail older patients with acute pericarditis, the application of guideline recommendations should be guided by the principles of geriatric cardiology rather than by a disease-centred approach alone. Therapeutic decisions must be individualised according to biological age, frailty status, comorbidity burden, renal function, and polypharmacy, integrating ESC guideline recommendations with comprehensive geriatric assessment and clinical judgement. Importantly, current ESC guidelines are largely based on evidence derived from younger and clinically stable populations, with frail older adults being underrepresented or excluded from pivotal clinical trials. Therefore, a significant evidence gap persists regarding the safety, tolerability, and effectiveness of standard pericarditis therapies in the elderly. This case underscores the limitations of extrapolating guideline-directed treatment to frail older patients and highlights the urgent need for dedicated research to develop age-adapted, patient-centred management strategies for acute pericarditis in older populations.

## Data Availability

The datasets presented in this article are not readily available because of ethical and privacy restrictions. Requests to access the datasets should be directed to the corresponding author/s.
