# Supplementary figures and images for: Case Report: Pleuro-myopericarditis in a frail older patient in an acute geriatric unit: the evidence gap in guideline-based therapy
Source: Front Cardiovasc Med. 2026 May 18;13:1798612. doi: 10.3389/fcvm.2026.1798612 (PMC13223131; doi:10.3389/fcvm.2026.1798612)

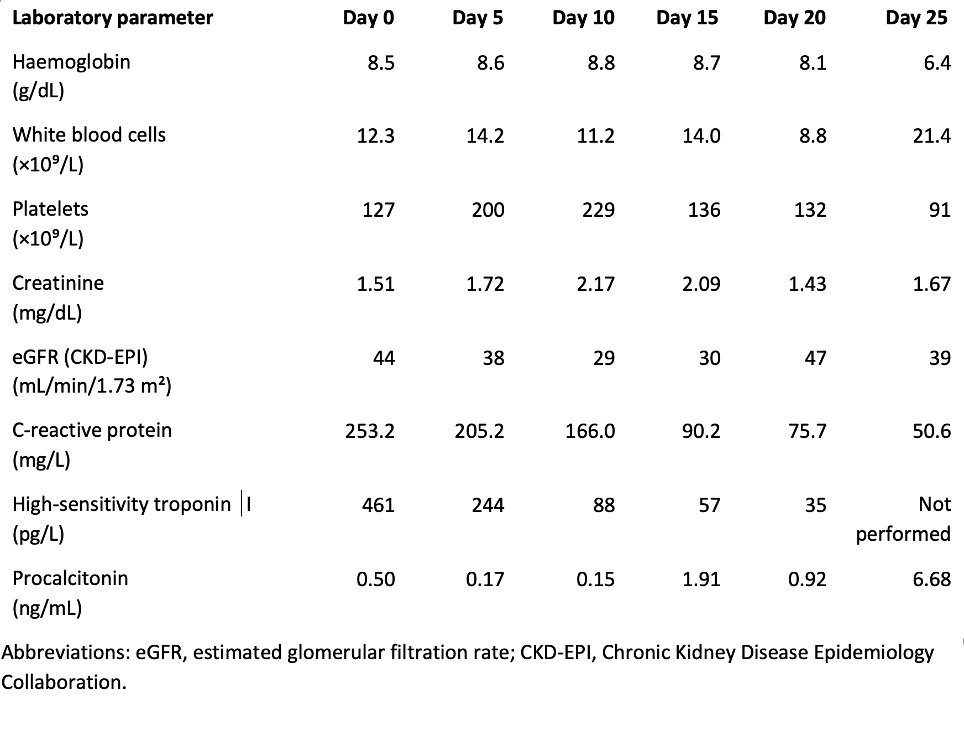

Supplement: Supplementary file 1 [file Image1.jpeg]

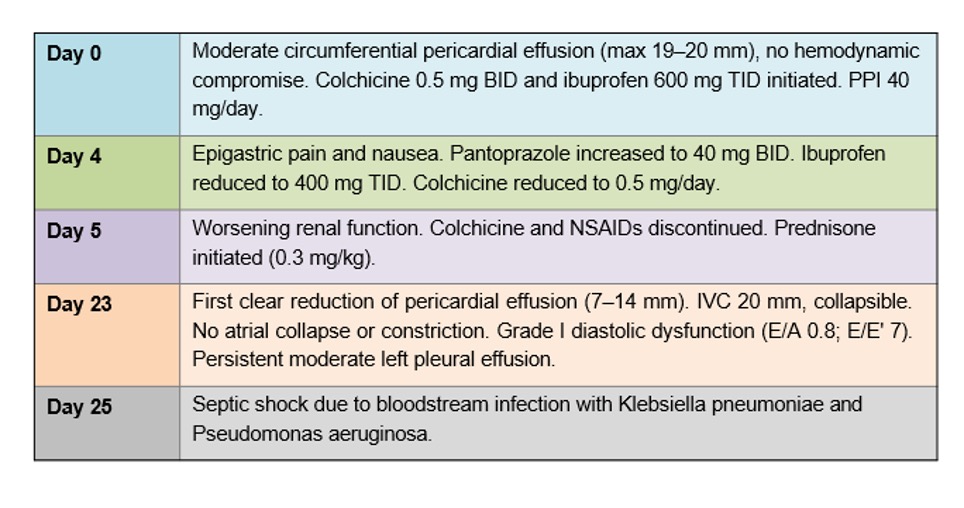

Supplement: Supplementary file 2 [file Image2.jpeg]
